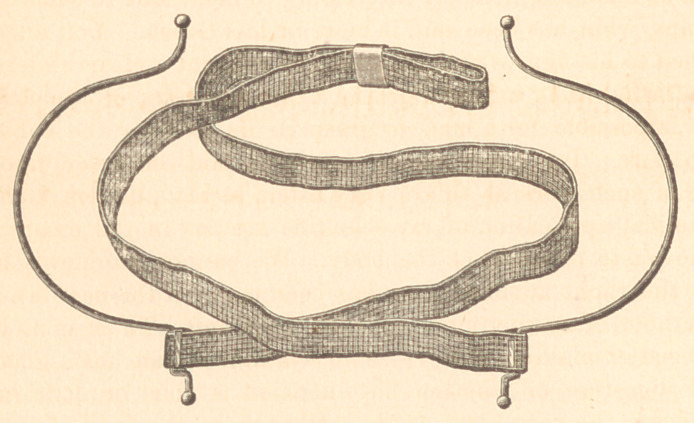# Massachusetts State Dental Society

**Published:** 1890-01

**Authors:** 


### Reports of Society Meetings.

### MASSACHUSETTS STATE DENTAL SOCIETY.[1]

### BRIDGE- AND CROWN-WORK THIRTY-FIVE YEARS AGO.

BY D. C. ESTES, D.D.S., LAKE CITY, MINN.

WITHOUT circumlocution, and with a desire to tax the time of this convention as little as possible, I ask your attention to the following very briefly stated facts:

In 1853 I was a student in the dental office of one Dr. Dumon, in the city of Albany, N. Y., and, after a few months of study and practice, was able to do about all the mechanical work connected with the office.

My preceptor was a skilful operator, a very close-mouthed, shrewd business-man, and had, for those days, a large practice. He advertised very extensively, and often boasted in the *press* of the amount of work he was doing; and in consequence of this course he was, so to speak, an isolated dentist, having but little communication with the local members of the profession. In the insertion of artificial dentures he was as proficient and as successful as any man I have since known, and resorted to about all the methods known to the profession of to-day. What is now known as bridge-work I know he practised, for the mechanical part of the work I did with my own hands, he alone directing and doing the adjusting. I supposed the method was common, and gave it no more attention than other kinds of work, and it was some years after I left his office that I learned that it was, perhaps, original with him.

Some three years after this time I became intimately acquainted with Drs. Douglas, Wood, and John Austin, who were then practising in Albany, and I made the method known to them. I am quite positive that Dr. Austin did some work of the kind, and perhaps the others did. All these men, together with my old preceptor, have, I believe, long since passed away.

I will take time to describe only two pieces of bridge-work

---

[1] Semi-annual meeting, Boston, June 6, 1889. The printing of the proceedings has been unavoidably delayed on account of non-receipt of papers.

which was done in this office. I do not remember what name he gave the work,—probably no particular name.

*First Case.*—The insertion of the right inferior cuspid and the adjoining bicuspid. The root of the cuspid remained, while the bicuspid had been extracted. The root was first carefully treated, drilled, and shaped, an impression taken, models made, dies cast, and a gold plate about one-quarter of an inch wide swaged to fit over root and all. Then a hole was punched in the plate directly over the root-canal, as indicated by the impression, a gold pivot inserted and firmly soldered. A short clasp to partly grasp the second bicuspid was then attached, after which the trial was made, and the articulation perfected in the usual manner. Common, plain plate teeth were then backed and soldered to the plate or bridge. The final adjustment was accomplished by partially filling the root-canal with amalgam and pressing the pivot and plate firmly into place. Just above the gold clasp, on the second bicuspid, a cavity was drilled, and a projecting gold filling inserted to keep the clasp and that end of the plate firmly and permanently in place. This job was a beautiful piece of work, and, as far as I know, successful in every respect.

*Second Case.*—A young man had had the two central superior incisors knocked out and the two laterals broken off. The roots were excised, nerves extracted, drilled, and otherwise properly shaped, and then, as in the first instance, a bridge or narrow plate, with metal pivots, constructed and inserted precisely as in the preceding case, only there were no clasps on the adjoining teeth. The metal pivots were slightly notched, but not, strictly speaking, barbed, though answering the same purpose.

I now pass to consider the methods practised by my preceptor for the insertion of what was then termed pivot-teeth, but was really crown-work then as much as it is to-day.

In the first place, every root was thoroughly treated and medicated, no matter what the time and labor required. Creosote was his great medicament, and in his hands appeared to be really a specific, for with it he cured all ulcers, so that, after the insertion of a pivot, no evil effects were discernible. The thorough preparation of the root was the secret of his success, for to solidly attach the crown was much more easily accomplished. The same for many years has proved true in my own practice.

Both wood and metal were used as pivots, which were cemented in place with either amalgam or gutta-percha, or what was then known as "Hill's stopping." To use the latter, the pivot was well warmed and enveloped in it, and while in a soft state inserted in the prepared canal of the tooth, and then pressed or driven home. In case of a badly-decayed root, all the carious parts were first removed, then the root properly medicated (ulcer cured) and drilled, after which a temporary polished brass pivot was inserted. Around this, amalgam was firmly packed with fine instruments, and the lost parts of the root built up until a firm base for the crown was secured. The slightly-projecting end of the temporary pivot was covered over with gutta-percha or wax, and the patient dismissed until next day, when the brass pin was removed, the root further shaped, and the crown finally adjusted with wood or metal pivot.

In cases where a thin tooth was required, on account of the peculiar occlusion of the antagonizing teeth, a gold cap was fitted over the root, a pivot soldered to this, and a plate tooth attached to the cap and then inserted in the same manner as described for bridge-work. I have practised this method myself and found it successful. Of course, all will remember that thirty years ago, and even later, all our crown-work was confined to the incisor and canine teeth, while we have at present crowns adapted to teeth with more than one root. However, before these were known, more than twenty-five years ago, I inserted my first artificial crown on a plurality of roots.

The roots of the first right superior bicuspid were drilled and shaped for base of crown, then a gold cap, as described above, was properly fitted, into this two metal pivots, corresponding exactly to the position of the two root-canals, and then a bicuspid plate tooth was backed and soldered to the cap, and finally adjusted in the usual way. I knew this case to have lasted more than twenty years, when I lost track of the patient. I have never since been able to do a more perfect piece of work, even with all our so-called modern improvements; and I may say that I have never seen a more serviceable job from the hands of any man. This, remember, is said for the method, and not the operator.

In conclusion, let me say that long ago I ought to have made the above facts known to the profession; but having six years ago lost all memoranda, all records, and most of my worldly effects by fire, I became, foolishly perhaps, completely disheartened, and resolved to lay aside the pen. Wiser resolutions, however, have prevailed, and now, with sight, nerves, and muscles unimpaired, I will join in with any young man of my age in the profession for a right-down lively race for the dental perfection. And God grant that we may not be called to cross the *bridge* and to receive the *crown* until we have fully attained unto this perfection.

### HEALTH IN THE OFFICE.[1]

BY H. B. NOBLE, D.D.S., WASHINGTON, D. C.

SINCE health is the corner-stone of all good work, either of body or mind, we venture on a few suggestions as to how this may be secured in our office-surroundings.

Pure, fresh air is of prime importance to health, yet how little value seems to be attached to it, if we may judge from the close unhealthy atmosphere of many of our dental operating-rooms, charged with the concentrated emanations from iodoform, creosote, and half a dozen more ill-smelling compounds, and mixed with the perfumes of dead pulps and foul teeth. Though the operator may exist in these improper surroundings for a long time, yet nature, insulted by such treatment, finally rebels, and protests in the form of headaches, backaches, weakened eyes, and the like.

To secure a healthy office we must have good light and good ventilation.

In regard to light, north, south, and east each has its advantages and disadvantages.

The north is a clear, steady light, but is not strong; and in the short winter months and cloudy weather its defects are clearly seen and felt.

The east is a good morning light, but is weakest in the closing hours of the day, just when one is tired and wants the best light possible.

A south light is the strongest and longest, and, if properly regulated by white curtains in the middle of the day, the best.

A west light should never be chosen if either of the others can be had, as it will be weak in the morning and bad in the afternoon, even if you are protected from the direct rays by a necessary curtain.

The dental operating-room should not be a small one, or a box partitioned off from an ordinary room, without apparent thought of air or ventilation. This condition of affairs is often seen in our large cities, sometimes several of these "stalls" being found in one end of a room. On the contrary, the operating-room should be of good size and separate from the parlor, or anteroom. The laboratory

---

[1] Read at the semi-annual meeting of the Massachusetts State Dental Society, Boston, June 5, 1889.

should be a commodious, light, sunny room, not the little back pantry-closet or dark, damp cellar often seen.

Sun and air must be had if either good health or good work is expected.

Then have all medicines in glass-stoppered bottles, in a case, so as to keep the office as free as possible from disagreeable odors.

It is not at all necessary to have one's office, instruments, or person so saturated with creosote and iodoform as to advertise one's calling. This is neither gentlemanly nor agreeable. No professional man is so likely to neglect proper exercise as the dentist, with his tired back calling him to the lounge or easy-chair; but air and exercise must be had, either by riding, driving, or walking. To this end it is well to have the office separate from the dwelling, so that in walking back and forth the eye may be allowed to take in fresh scenes and the lungs fresh air, thus refreshing both mind and body.

DISCUSSION ON DR. NOBLE'S PAPER ENTITLED "HEALTH IN THE OFFICE."

*Dr. Geo. F. Waters.*—Some time ago I tried to solve the question as to which was the best kind of light, whether the direct or the reflected rays of the sun. The first thought that came to me was, What action does sunlight have on the body? I had a double convex lens, silvered on one side, and with this I discovered that a ray of sunlight striking on my hand or foot gave tonicity to the body; it caused a contraction of nerve-fibres. After discovering this, I mentioned the matter to a medical friend of mine, who was rather sceptical, but still open to demonstration. I told him to take the glass and get a focus, so that he could see the action of the iris. He held the glass, and with a mirror I threw the light on the back part of his head. I threw it on his hand, and he said, "I think I can see some of that light." I took a book, and thoroughly protected his eyes with it, and he found that whenever I threw the sunlight upon the surface of his body the action of the eye told it at once. I have come to the conclusion that sunlight upon the skin —exposed a long time—causes arterial tension. This brings on headache and meningitis,—sunstroke, which is nothing more than apoplexy,—relief from which is obtained by bringing the blood from the brain back to the heart. I concluded that for my eye a north light would be best. I experimented some with some tadpoles that were just hatched out,—little fellows,—and part of them I put in a closed vessel with some plants, and set it in the sunlight. A similar vessel, filled in the same manner, was placed in the north light. Both vessels were filled with the same kind of water; and the result was that the tadpoles that were placed in the north light grew to be very large fellows, while those placed in the sunlight became frogs while they were very minute,—perfect frogs, only one-half inch long. That was the tonicity of the sunlight contracting the cells.

*Dr. J. N. Crouse.*—Are you sure, doctor, you had the same species?

*Dr. Geo. F. Waters.*—There was no doubt about it. My reason for selecting the north light was that, in a majority of cases, when persons use light on their work they prefer the north light. It is the case with engravers. They never allow the sun to shine upon their work, but want the reflected north light. The essayist's idea of the size of the room is good, although I do not care to have a room so large that any number of persons, who are not interested in the work going on, can press in and crowd upon the chair.

*Dr. V. H. Jackson.*—The doctor did not tell us what effect the sun shining upon the hand had,—how it acted on the iris.

*Dr. Waters.*—It acted on the iris so as to contract the pupil.

*Tuesday, June 4, 1889.—Evening Session.*

The evening session was devoted to a lantern exhibit by Dr. W. Xavier Sudduth, Philadelphia, illustrating "The Nature of Formative Cells."

DISCUSSION ON DR. SUDDUTH'S LANTERN EXHIBIT.

*Dr. R. R. Andrews.*—I am somewhat familiar with these different processes, as you know, and I must say that I never saw such a fine exhibition as the one given to-night. It is something we may remember all our days, and my only regret is that we have not the room full of physicians as well as dentists to appreciate this work. The coloring of the slides is another important feature: it rests the eye. Some of them are opaque and not clear, but with a little more careful work could no doubt be made quite as clear as the others. I think it is an improvement on our old method of preparing them. There is only one fault in it that I see, and that is, you have to take a section that covers the entire field.

In my appreciation of the work I feel much as an old gentleman did some years ago. His son asked his advice regarding some matter, when he answered, "I cannot advise you,—you are out of my reach." Dr. Sudduth is so far out of the reach of most of us that all we can say is a good Methodist "Amen."

Another point of interest and importance was the slide showing the vascular supply of the pericementum and of the pulp of the tooth. They are more intimately connected than we generally suppose. I had taken for granted, as I presume every one here has, that there was an arterial supply of considerable size that went into the foramen of the tooth,—some to the pericementum, and some to the pulp. From the showing, however, it seems that we have to proceed through a third party when we speak of inflammation of the pulp producing inflammation of the pericementum. I suppose the nervous supply is made up in a similar way; consequently, it is easier now to understand how the peculiar sensitiveness of the pericementum from the irritation of the pulp occurs, and why the irritation so quickly projects itself into the pericementum.

*Dr. W. X. Sudduth.*—I will add, that, if you will take ground sections of cat's teeth you will find out how many foramina these teeth have for vascular supply. I once had a case in practice where a central incisor was lost because of pericemental absorption, caused by an application of arsenic in the root-canal. After the root was prepared a crown was placed on and worn for two weeks, after which time the patient came back and had the root removed. I found considerable absorption of the root. I studied it carefully, and found a large foramen on the side of the root. It was through that foramen the arsenic had passed out and given rise to the trouble. In studying the anatomy of teeth, and of which we do not do enough, you will find many of these points brought out. It is important that students should be made to have a good knowledge of the anatomy of the teeth, for when they get in practice then they have little time for study.

*Dr. C. T. Stockwell.*—I wish to add my personal emphasis to everything that has been said in the way of appreciation of this lecture. There was one thing that constantly ran through my mind, and that was, "Function precedes organism." I wish to ask Dr. Sudduth if he has any criticism to make upon that scientific postulate. It seems to me that in a general sense it has an immense physiological meaning.

*Dr. Sudduth.*—If you mean that there is functional activity before there is organization of tissue I should say no, because all tissue is organized in one sense, but if you mean that functional activity exists before organs are developed, then I should answer yes, as many forms of life perform function without any regularly developed organs.

*Dr. H. C. Merriam.*—Some years ago I was interested in this subject, and I got the impression very strongly that function, according to Spencer, was not only a determining cause of structure, but that function preceded it.

*Dr. Sudduth.*—Only in so far as it is revealed in hereditary tendencies. Each individual cell has in it the principle that will cause the development of organs or, in other words, organization.

*Dr. Andrews.*—In health or disease!

*Dr. Sudduth.*—According to the hereditary tendency of the pre-existing cell, or according to the peculiar influence, normal or pathological, which surrounds the developing tissue. Cells as well as individuals are subject to environment.

*Wednesday, June 5, 1889.—Afternoon Session.*

NEW PREPARATIONS.

*Dr. W. X. Sudduth.*—In my contact with the manufacturers, I am always on the look-out for new points of interest to the dental profession, and a year ago I presented to you an article, the silico-fluoride of sodium. It is non-poisonous, and can be given internally in doses of from five to ten grains three times a day without any injurious effect. That it prevents acid fermentation is also an established fact. Since I presented it, a year ago, it has been in constant use by the manufacturers of syrups for soda fountains, as it keeps them perfectly sweet. Lately, there has been a successful effort made to prepare them in tablet form, and, by the addition of the essential oils, to prepare a substitute for carbolic acid for use in the mouth. These now come in tablets, so that one dissolved in two ounces of water gives you the right strength to use as a stimulant antiseptic mouth-wash. This is a very convenient form for use. They can be procured from H. K. Mulford & Co., of Philadelphia.

PRESENTATION OF NEW INVENTIONS.

*Dr. Horatio C. Merriam.—The Ives Dental Syringe* (C. F. Ives, M.D.S., New York City).—The special feature in this is the improved packing. Instead of its being made like the ordinary syringes which makes it so difficult to use acids or any strong medicines, this has a piece of hard rubber with a feather edge, causing it to perfectly fit the barrel, which is carefully ground. Strong acids nor any of these medicines have any action upon it. The points are made of platinum and iridium, and the syringe is applicable for all purposes where a small syringe can be used.

*Dr. W. H. Jones*, Northampton, Mass.—*New Forms of Excavators.*

*Dr. E. C. Blaisdell*, Portsmouth, N. H.—*Model of Instrument-Holder.*—The feature is that when the drawer is drawn out the instruments, which cover a spring, are lifted into view. Upon shutting the drawer the spring is pressed down and the instruments are stored away again.

*Dr. H. W. Gillett*, Newport, R. I.—*An Appliance for separating Teeth to hold Matrices, etc.*—Also a bracket table to go on the S. S. White bracket, which is large enough for a majority of instruments used,—the excavators on one side, the gold-filling instruments on the other,.which shows what can be done in the way of getting instruments into a compact and readily reached position. Another appliance is a little instrument that will not slip, but hold the teeth firmly in getting space. It consists of a small piece of piano wire, with a thread cut upon it. One end is flattened, and on the other end is a little nut. A small washer goes on the other side between the teeth. It is a matter of considerable convenience, and in some cases a very serviceable little separator. It is not applicable to cases where it is liable to slip against the gum. That may be modified by putting a little sand-paper washer under the nut.

*Dr. Kirk A. Garland*, South Boston, Mass.—*Gas-Bracket for heating Cases before soldering.*

*Dr. Horatio C. Merriam.*—*New Bracket and Socket Handle.*—The handles are not mine, but the invention of Drs. Perry and Darby. We formed a club and had some made. They are very strong, and have a long taper. You will see the security with which the instruments pass in. One turn tightens them. Many like this sort of an instrument, and they are also suitable for holding a mirror where you want a smooth handle.

The other is a new table, which I present to the profession. My object was to secure a table of plain and substantial workmanship, where the expense of manufacture was gotten rid of largely by avoiding all curves and ornamentation, and also in such a way that it gives the most space for the handling and working of instruments. It works from two sides. The drawers can be divided into halves,—one part being a receptacle for instruments used in preparing the cavity,—and by pushing it in and out of the way you have brought out on the other side the instruments necessary for filling or finishing the cavity. The covering of the table is of fancy paper,—shellaced. This can be kept clean by wiping it off with a sponge. The lower drawers are made for burrs, etc., but these can be improved so as to hold a larger quantity.

I am just making some studies upon a subject entirely neglected by the profession. As far as I learn, very few dentists have made a study of files for the profession for use in an anatomical way. My object is to find a file that I can use at the cervical wall, having a safe border which can be rolled back and forth without injuring the gum or rubber dam, and made of such a quality and workmanship that they will be serviceable instruments to us. These files are now rights and lefts, but I am having other forms made. These are professional files,—for the profession, and never will pass into the hands of combination dealers. They are placed outside where all can have an equal opportunity for enjoying their manufacture and sale. If you think the files I have given you are of service to the profession, and you can make use of them, and also the table, I shall be glad to have you accept them.

*Dr. B. H. Teague*, Aikin, S. C.—The *impression material* I have here is a compound I have used for the past ten years. The advantages are that it is fragile,—easily broken from the mouth, and of great benefit in taking impressions when overhanging teeth make it difficult to remove the ordinary material without destroying its shape. It is a material you can cast melted zinc into by making a proper mould. This can be done by encircling the impression with an iron ring, and pouring sufficient of the material around it to form a mould. It is a splendid material for soldering bridge-work. It is also very easy to detach the impression from the plaster cast. This impression was taken from a model I had made to construct a set of teeth upon. With the exception of one or two little breaks the impression is quite ready for another case. This material is patented.

This appliance—*an arm-rest*—has been in use for at least five years, and it has proved an infinite source of satisfaction to myself. Many times I have been tired out in operating, and I could find no help for it unless I put half of my body on the chair, or encircled the head of the patient, or had a head-rest that resembled a pulpit more than it did a dental chair; so I devised this fixture, which is swung from the ceiling, or wall, and by slipping my arm through it and resting my body upon it at times, it is a great source of relief.

Another thing, in operating for a great many sick people, as I have to in Aiken, I find that I get too near them, for their breath is often very offensive, and this enables me to get a little farther from the head of my patient, and at the same time hold my hand and arm steady. An additional advantage is you are able to make examinations of ladies' mouths without troubling them to take off their hats. It is very easily arranged and you can slide it up and down, and when a person learns how to use it he will never be without it.

CLINICS.—ORAL SURGERY.

*By Dr. G. L. Curtis*, Syracuse, N. Y.—1. *Removal of Necrosed Bone due to Alveolar Abscess.*—The patient, a young man, had two superior central incisors pulpless and abscessed, and for whom a dentist had practised immediate root-filling. A fistula led to the apex of the right root, while the abscess of the left root was not indicated by an external opening, and, in consequence, was styled a "blind abscess." The opinion of the operator was that there existed a subperiosteal opening leading to the fistula over the right root.

The operation consisted in passing a rose-head burr, of suitable size, into the fistula, and following along its track into the excavation in the maxilla, about half an inch in size. The dead necrosed bone surrounding the cavity, including the sack attached to the end of the root, was burred away, after which the cavity was carefully syringed with tepid water, and all dislodged diseased tissue washed out. A few drops of aromatic sulphuric acid were then injected into the cavity with the view of destroying any diseased tissue remaining and stimulating the parts to healthy action.

The "blind abscess" was opened into directly over the end of the root by means of a spear-pointed drill, and the diseased part cut away and treated as in the case of the right central. A twenty-five-per-cent. solution of cocaine was employed, reducing the pain to a minimum. The operation was completed in ten minutes.

2. *Transplantation.*—Patient, a boy thirteen years old. Five days previous to clinic he fell, knocking out his superior left lateral incisor, which was lost.

The overlapping gum was dissected away, and a spiral knife used to cut out the granulations which filled the socket, which was afterwards cleansed and sterilized. The tooth, which was previously prepared and sterilized by Dr. Fillebrown, was then placed in position, and retained by means of clasps attached to approximating teeth. The gum surrounding the central incisors was much inflamed and the teeth quite loose and tender to pressure, consequent upon the original injury.

3. *Dentigerous Cyst.*—Patient, a man twenty-seven years of age. The lesion was due to the non-eruption of an inferior right cuspid. The case was mistaken and treated for alveolar abscess six weeks previous to the clinic, which operation consisted of simply lancing the gum over the tumor. The tooth was found to be parallel with the jaw, deep in the substance of the bone, and around it the alveolar wall was necrosed to a considerable extent, the disease extending past the symphysis as far as the left cuspid, where an opening appeared in the gum. Between these points (the right second bicuspid to the left first bicuspid) the soft tissues were completely detached from the jaw, and, to a marked degree, hypertrophied. The patient experienced no pain from probing while the case was being demonstrated.

4. *Bridge-Work.*—Dr. Curtis demonstrated his removable bridge, the model being a bridge composed of the superior left second bicuspid and first molar, attached to the first bicuspid and second molar. The attachment was by an arm extending from the second molar and first bicuspid over which a sleeve, attached to the bridge, was slipped, and which locked the teeth together and prevented movement. The case is fastened by chloro-percha, and is not intended to be removed by the patient. It can be kept as clean as the full soldered bridge, and a marked advantage is, it can be applied to teeth in any position, and does not necessitate the cutting away of the teeth until the walls are parallel with each other. The bridge was well made, and admitted by all to be the most complete and practicable of its kind now in use.

*Dr. Thos. Fillebrown* demonstrated the quick preparation of proximal cavities without separation, using a left superior central for the purpose; also a superior method of attaching the rubber dam.

*Dr. T. H. Parramore,* Hampton, Va., demonstrated his method of capping with sterilized sponge, the case being an inferior right lateral incisor, so far exposed that the nerve could be plainly seen. The sponge was applied, and the cavity filled with oxyphosphate. After allowing six months to elapse the oxyphosphate, excepting a thin layer over capping, is to be removed and gold substituted.

*Dr. S. C. G. Watkins,* Montclair, N. J., gave a clinic, the operation being the filling of an inferior second molar with copper amalgam, using his trimmers and special instruments.

*Dr. W. H. Pomeroy,* Gloucester, Mass., filled a right superior molar, masticating surface, by a method similar to Herbst's, and using soft pellets worked by his patent mechanical engine mallet and smooth burnishers.

*Dr. J. F. Adams,* Worcester, Mass., demonstrated a method of filling and contouring by means of hand-mallet, using Williams's crystalline gold, the case being a superior left canine tooth.

*Dr. G. L. Chewning*, Fredericksburg, Va., exhibited a model of fracture in three places of the inferior maxilla that had been successfully treated by him. The feature was, to take the impression while holding the parts in their normal position instead of securing the impression as the fracture exists, and then breaking the cast and adjusting to the proper relation.

*Dr. C. C. Carroll*, of the Carroll Aluminium Manufacturing Company, New York City, exhibited and demonstrated practical cases, artificial as well as crown- and bridge-work, made of aluminium, and also introduced a non-cohesive aluminium foil, new to the profession, for the purpose of filling teeth.

*Dr. V. H. Jackson*, New York City, exhibited a large number of models of cases of irregularities that had been successfully corrected by him by means of his various appliances for the purpose.

*Dr. Joseph E. Waitt*, Boston, Mass., demonstrated a method of making mouth-mirrors by which the cost was reduced to a minimum. It consisted of making three dies, each with a round hole, the first for shaping and the others for finishing the backing for the mirror, which backing is made of German silver and stamped into the die by a steel mandrel. The mirrors are cut by a diamond glass-cutting machine, and cemented into the backing. This method is not patented, and was exhibited for the benefit of the profession.

*Dr. Waitt* also exhibited a nitrous oxide light for use in making photo-micrographs and also as a light for stereopticons.

*Mr. E. T. Wetzel*, Basel, Switzerland, illustrated his manner of winding springs from piano wire for dental engines and other purposes.

*Dr. S. S. Stowell*, Pittsfield, Mass., demonstrated his method of making and setting crowns. The root is prepared in the usual way and the pin and cap of metal carefully measured and adjusted. The pin in the crown, if it be a Logan, is next cut off, with the exception of a small piece that is allowed to remain. The crown is next ground and fitted to the cap over the root, after which it is removed and gold melted into the undercut and around the pin, the metal being spatted down while in a state of fusion, which forces it to every part and produces a flat surface. The crown is again ground to fit the cap, and afterwards soldered to it, thus producing the most cleanly crown it is possible to make. It is cemented to the root in the usual way.

*Dr. George A. Maxfield*, Holyoke, Mass., showed an ingenious way of making hard rubber corundum disks. The corundum grit is incorporated with the rubber by being passed through a clotheswringer (the quantity of the mineral used influencing the coarseness of the disk), and, after being cut to the required diameter by a mandrel for the purpose, they are vulcanized, being strung on a fine wire, the diameter of the engine mandrel, and separated from each other by a small circular piece of tin, the whole encased in a clamp to press and hold them together. A great many can be made at one time, and at a cost that is not worth mentioning.

*Dr. A. Ritter*, Utica, N. Y., exhibited an engine standard of his own construction, the propelling power being furnished by a one-tenth horse-power motor. This was covered with a box which greatly reduced the noise, and was controlled by a switch that would start and stop it instantly. The cable could be detached at will and any other light mechanism run in its stead. The contrivance also included a fan and a corundum wheel for grinding, all running at the same time. It was an ingenious arrangement and invaluable in the many advantages it contained. The motor can be run by power from a street wire.

*Dr. A. H. Brockway*, Brooklyn, N. Y., demonstrated a method of applying the rubber dam.

*Dr. W. S. Elliot*, Hartford, Conn., exhibited a new *rubber-dam holder and separator.* Simplicity and efficiency are qualities always desirable in any instrument or appliance that is called into daily use. Among those that seem to be capable of improvement is the rubber-dam holder. The well-known Cogswell instrument has served exceedingly well a general purpose; but to smooth out the wrinkles and folds of the dam something different was needed, and this need is abundantly supplied by a new device, of which the cut is a fair representation. The dam is first adjusted to the teeth, then over each cheek, the folds are smoothed out, and while the spring of the holder is slightly compressed the rubber is made to stretch over the rings at the ends of the wires. Upon releasing the spring the rubber is caught and held firmly as the braid is passed over and adjusted behind the head.

The exhibition of goods by the dental dealers is deserving of special mention. An arrangement of this kind has a twofold and mutual benefit, the society being able to examine in one building the latest novelties and appliances of interest and use to the profession, while the dealers have the advantage of reaching a large number of men at one time. The present exhibit was fully in keeping with those of former years, and the executive committee are to be congratulated on the success that attended their carefully-laid plans and unremitting efforts.

## Figures and Tables

**Figure f1:**